# Pragmatic Applications and Universality of DNA Barcoding for Substantial Organisms at Species Level: A Review to Explore a Way Forward

**DOI:** 10.1155/2022/1846485

**Published:** 2022-01-11

**Authors:** Sarfraz Ahmed, Muhammad Ibrahim, Chanin Nantasenamat, Muhammad Farrukh Nisar, Aijaz Ahmad Malik, Rashem Waheed, Muhammad Z. Ahmed, Suvash Chandra Ojha, Mohammad Khursheed Alam

**Affiliations:** ^1^Department of Basic Sciences, University of Veterinary and Animal Sciences Lahore, Narowal Campus, 51600 Narowal, Pakistan; ^2^Department of Biochemistry, Bahauddin Zakariya University, Multan 60800, Pakistan; ^3^Center of Data Mining and Biomedical Informatics, Faculty of Medical Technology, Mahidol University, Bangkok 10700, Thailand; ^4^Department of Physiology and Biochemistry, Cholistan University of Veterinary and Animal Sciences, 63100 Bahawalpur, Pakistan; ^5^Subtropical Insects and Horticulture Research, Agricultural Research Service, U.S. Department of Agriculture, 2001 South Rock Road, Fort Pierce, FL 34945, USA; ^6^Department of Infectious Diseases, The Affiliated Hospital of Southwest Medical University, Luzhou 646000, China; ^7^Department of Preventive Dental Science, College of Dentistry, Jouf University, Sakaka 72721, Saudi Arabia; ^8^Department of Dental Research Cell, Saveetha Dental College and Hospitals, Saveetha Institute of Medical and Technical Sciences, Chennai, India

## Abstract

DNA barcodes are regarded as hereditary succession codes that serve as a recognition marker to address several queries relating to the identification, classification, community ecology, and evolution of certain functional traits in organisms. The mitochondrial cytochrome c oxidase 1 (*CO1*) gene as a DNA barcode is highly efficient for discriminating vertebrate and invertebrate animal species. Similarly, different specific markers are used for other organisms, including ribulose bisphosphate carboxylase (*rbcL*), maturase kinase (*matK*), transfer RNA-H and photosystem II D1-ApbsArabidopsis thaliana (*trnH*-*psbA*), and internal transcribed spacer (ITS) for plant species; 16S ribosomal RNA (16S rRNA), elongation factor Tu gene (*Tuf* gene), and chaperonin for bacterial strains; and nuclear ITS for fungal strains. Nevertheless, the taxon coverage of reference sequences is far from complete for genus or species-level identification. Applying the next-generation sequencing approach to the parallel acquisition of DNA barcode sequences could greatly expand the potential for library preparation or accurate identification in biodiversity research. Overall, this review articulates on the DNA barcoding technology as applied to different organisms, its universality, applicability, and innovative approach to handling DNA-based species identification.

## 1. Introduction

DNA barcodes represent short gene sequences that are drawn from a standardized part of the genome and can be used as a unique identification marker to recover and characterize species. As such, DNA barcodes are a vital resource and an innovative molecular diagnostic tool, as has been demonstrated in recent decades [1–3]. DNA barcodes can identify unknown samples by matching a specific genetic marker to a reference sequence library [1, 3, 4]. Ideally, DNA barcodes have low intraspecific and high interspecific distinction [5]. Short gene sequences can easily be taken from the vouchered specimen and have successfully been used to distinguish species as well as populations [6]. Identification of species is carried out by amplifying highly variable regions, for instance, DNA barcode region of either nuclear, chloroplast, or mitochondrial genomes using polymerase chain reaction (PCR) [7]. Methods that are applied in DNA-based identification systems are based on standard molecular biology techniques. The laboratory method includes extraction of DNA, PCR amplification, and identification by DNA sequencing following sample selection and documentation. At the same time, data management involves sequence alignment and assignment of barcode IDs to sequence for further identification. The National Center for Biotechnology Information (NCBI) provides a large suite of online resources for biological information and data for subsequent investigation. The sequence is retrieved from a publicly available NCBI database (http://www.ncbi.nlm.nih.gov/gene/) of annotated genomic, transcriptomic, and protein sequence records. The alignment is performed using different software such as BLAST and Clustal W, which precisely distinguish species by comparing their DNA sequences to those of known sequences as presented in reference libraries [2, 4, 8–10]). Various software has successfully been used to collect and store DNA sequences along with DNA barcoding data of different organisms (Table 1).

**Figure 1:**
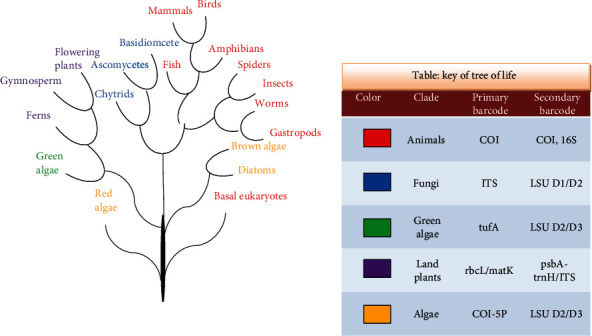
Tree of Life with some of discovered primary and secondary code barcodes. DNA barcoding in conjunction with morphological, ecological, and biochemical information reveals an intriguing diversity of species in discovering and describing new species for fern, algae, fungi, bacteria, viruses, arthropodes, nematodes, mollusks fish, amphibians, reptiles, birds, higher plants, and mammals.

The prime goal of establishing DNA barcoding is to construct a library of each single species that are present on earth [11] (Figure 1). Although the use of DNA sequences for biological identifications is not new, however, the concept of “DNA barcode” as a reliable and definite identification method of all forms of life (plants, animals, fungi, bacteria, and viruses) has great promise [12]. A short mitochondrial gene that encodes cytochrome c oxidase 1 (CO1; 760 bp) has been reported as the standard and practical DNA barcode for the identification of many animal species. DNA barcodes used for plants include plastids (e.g., *rbcL*, *matK*, *trnL-F*, *trnH*, *psbK*, *and psbA*) with nuclear ITS spacer, while nuclear ITS for fungi and 16S rRNA, *Tuf* gene, and chaperonin for the bacterial strains [7, 13].

In asexually reproducing organisms, any of the gene sequences can be used as a barcode to identify a particular species of interest. DNA barcoding helps to reveal pertinent information about the hereditary as well as evolutionary interactions through the integration of molecular, morphological, and distributional data [14]. Barcode investigation framework can shed light on hereditary variations in chronic disorders. Furthermore, barcoding distinguishes the single-point mutation inside exons [14].

DNA barcodes can be applied as tools to address fundamental questions in evolution, ecology, and conservation biology. The use of DNA barcodes has attracted widespread interest in recent years, and it offers exciting prospects for use as a new taxonomic tool. Thus far, DNA barcoding has been employed for a diverse array of applications as will be described hereafter. Firstly, DNA barcoding tools have boosted our potential to identify potential targets without undue discomfort to animals and invasive sampling procedures, which can be challenging to study with conventional methods. Continuing with this, barcoding further assists in forensic testing, for example, utilizing hair, blood, and waste materials. It exhibits great significance in the forensic investigation due to its nonintrusive technique to distinguish living specimens [15, 16]. DNA barcoding has been regarded as the best strategy for segregation of commercial food products by the United States Food and Drug Administration (US FDA) [17]. It is additionally relevant for species identification in case of dead and degraded specimens when morphological characterization is in critical condition [18–21]. The intra- and interspecific differences taken from DNA barcoding help us enormously in deciding evolutionary history and relationship among species [14, 22, 23].

Historically, DNA barcoding has been claimed to be a revolutionary and innovative approach for the identification of living organisms. But, the introduction of novel methods for analyses in scientific research often brings controversies and concerns. Hence, initially, DNA barcoding also faces the same fate in the field of taxonomy [24]. However, DNA barcoding can help remedy the field misidentification, helps to make species identification more accurate, creates open-access databases, and expands technical expertise by allowing taxonomists to accurately sort samples and by highlighting divergent taxa that may represent new species [11]. DNA barcoding has many advantages with criticisms raised against the ability to discover new species and its reliability. The primary focus of this review is to highlight the use of DNA barcoding for all wide range of tasks in the life sciences while also to demonstrate its values in each discipline and to discuss the reliability and prospects of DNA barcoding.

## 2. DNA Barcoding in Marine Life

### 2.1. Fish

**Figure 2:**
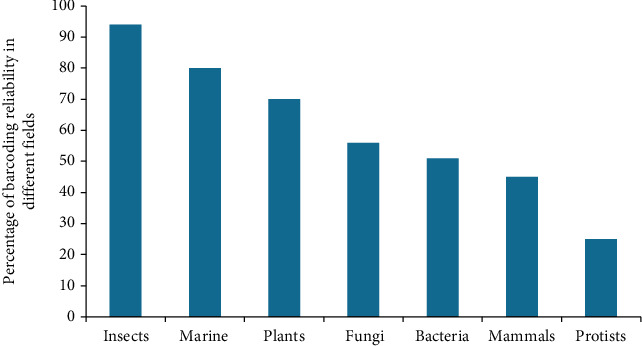
Barcoding reliability in different organism on the basis of percentage identification. Marine life depicts almost 80%, insects 94%, protists 25%, plants 70%, fungal life 56%, and bacterial species 51% reliable tool. The reliability of such procedure depends on an effective distinction between intra and interspecific variations, which is missing for several of the taxa studied for humans, as indicated by significant juxtaposition of sequences.

By using DNA barcoding, over 95% of aquatic species have properly been identified [14, 25]. This tool empowers the evaluation of entire fish, fillets, bout, fractions, juveniles, caterpillar, and ovum concerning aquatic life [26]. The viability of barcoding has been depicted by the superior identification of fish species with more than 90% of success rate. The specific barcode sequences from any segment could be coordinated against the reference collection with regard to the Barcode of Life Data System (BOLD) (http://www.barcodinglife.org). Firstly, cytochrome oxidase B was utilized as a barcoding region in organisms from land and water, but later, studies had exposed its limitations by declaring it as a nonreliable source of barcoding that was replaced by cytochrome c oxidase [2, 4]. The accuracy of species identification of Ponto-Caspian Alburnoides by DNA barcodes has been described to reach nearly 100%. Furthermore, one potentially new species within the A. gmelini species has been reported. Despite the limited ability of COI to infer phylogenetic relationships, a study had furnished a shred of evidence that the Ponto-Caspian lineage of Alburnoides includes a significantly larger number of species from the Caspian Sea basin and inland basins of Central Asia [27]. CO1 barcoding region additionally offered opportunities to discover fish caterpillar separated from the great barrier deep sea. More than 5000 fish species have been barcoded using CO1 [25, 28, 29]. To prevail in the differentiation power of fish species, sequences from four published studies on Australian fish were combined [30–33]. The consequences in 2005 clarified that the utilization of 16S rRNA and 12S rRNA has a higher potential than that of CO1 in assigning sequences to the level of classes and orders. This census had demonstrated that it has the additional standard marker for barcoding in all vertebrates and organisms from land and water, which may significantly help in phylogenetic reconstruction. Cryptic species such as marine diatoms and scavengers can be revealed by barcoding. Genetic analysis of partial mitochondrial CO1 barcode sequences of 473 specimens assigned to 52 morphological species of the genus *Trimma* fish had revealed the presence of 94 genetic lineages [34–36]. In April 2011, sequences from over 80% Canadian and American species were obtained by barcoding, and this consisted of 5624 fish species, 50 families, 178 genera, and 752 species. However, about 28% of freshwater fish (i.e., which will be threatened or endangered) still needs consideration of taxonomist [37, 38]. Similarly, certain parts just like fillets or bout whose morphological elements tend to miss demand molecular recognition [26]. Yet at the same time for the validation of fish genera, DNA barcoding is a helpful technique due to its superb reliability (93-98%) [39] as presented in Figure 2.

### 2.2. Marine Microbes

Appraisal of biodiversity in the microbial world has been a long, challenging task. Rapid and precise recognition and detection of microbes' areas are often necessary to prevent the spread of diseases brought on by microbes. Protists are eukaryotic microorganisms which have short era time and a biogenetic conceptive capability. A naturally noteworthy group of protists are the dinoflagellates which serve as important markers and are also the cause of red tides. DNA barcoding of marine ecological samples uncovered the enormous dinoflagellate diversity [40].

In particular, the natural diversity of dinoflagellates was investigated in three diverse marine environments (Northeast Pacific, Northwest Atlantic, and the Caribbean) in which the single-cell barcoding was used to identify uncultivated groups. From all three environments, the great majority of barcodes were not represented by any known cultured dinoflagellates. An explosion in the diversity of genera was also observed in which a modest number of known species belonging to Kareniaceae was already in existence. About 91.5% of nonidentical environmental barcodes represent distinct species. Still, only 51 out of 603 unique environmental barcodes could be linked to cultured species by using a conservative cut-off based on distances between cultured species. COI barcoding was successful in identifying species from 70% of cultured genera.

A scientific study on the identification of marine microbes was conducted in 2010. Out of 669 culture assemblage samples from 11 collections, 566 COI amplicons were recovered as some taxa failed to amplify (most commonly, these were *Amphidinium* sp., *Heterocapsa* sp., *Oxyrrhis* sp., and some unrevealed gymnodinioid dinoflagellates). In particular, 304 amplicons were successfully direct-sequenced with enough quality to act as barcodes. Of these, 293 amplicons were incorporated for barcoding analysis (the others being resolved to be nondinoflagellate sequences), together with 62 publicly available dinoflagellate COI sequences from Genbank. This developed to a total of 336 sequences, showing 54 named species and five Symbiodinium clades. Most culture collections were deliberately biased towards photosynthetic, planktonic, and toxic genera such as *Alexandrium* and *Scrippsiella* [40]. Tintinnid ciliates are thought to be suitable models to investigate the diversity and biogeography of microbial plankton. Phylogenetic resolution, biogeography, and heterogeneous diversity within and among tintinnid lineages had raise questions about the unique processes that promote their diversification and determination of their spatial distribution [41].

### 2.3. Marine Algae

DNA barcoding methodology as described by Hebert and colleagues has already revealed novel diversity in protist taxa using the COI marker including red algae, brown algae, diatoms, and the ciliate genus *Tetrahymena* [4]. Different types of red marine macroalgae are often troublesome to distinguish by utilizing morphological procedures. Two molecular markers specific to mitochondrial COI gene and Universal Plastid Amplicon (UPA) domain V of the 23S rRNA genes was used for recognizable proof of various types of red algae belonging to the family Kallymeniaceae. Moreover, COI was seen as a highly sensitive marker and had prompted the disclosure of another species Euthoratimburtonii [42].

A comparative study was conducted including intertidal red macroalgae in China with three molecular markers consisting of COI, UPA, and ITS. Although COI was shown to be robust for distinguishing the species yet not all species gave fruitful amplicons because of the absence of widespread primers. UPA compellingly had all-inclusive primers and finally showed issues for firmly related species, while it was least advantageous [43]. Gracilariaceae is a red algal family which is commercially predominant for its exploitation as a phycocolloid agar in biotechnology (i.e., its exploitation as a phycocolloid agar in biotechnology and microbiology research). *Gracilaria* species are troublesome to recognize morphologically, and DNA barcoding holds assurance in species-level recognition [44]. *Gracilaria mammillaris*, the most common flattened *Gracilaria*, has been reported from North Carolina offshore waters. Analysis of algal *rbcL* genes from North Carolina specimens identified as *G. mammillaris* discloses that they are *Gracilaria hayi*, *Gracilaria galatensis*, *Gracilaria occidentalis*, and *Gracilaria isabellana*. Comparison of contemporary *Gracilaria rbcL* sequences with the partial sequence from the *G. mammillaris* holotype showed that *G. mammillaris* is most likely not present in North Carolina. Specimens from Brazil and Venezuela initially identified as *Gracilaria curtissiae* are *G. mammillaris*, and the currently designated *G. mammillaris* epitype presents a unique species described here as *Gracilaria gurgelii* sp. nov. [45].

Recently, a novel microalga has biofuel potential and has been isolated and characterized from the Indian Ocean. In this work, conserved DNA regions of chloroplast genome corresponding to 16S and 23S rRNA were used as a barcode [46]. Both genetic markers were found to be near at the inverted repeat of the chloroplast genome, which could have less evolutionary changes with respect to a single-stranded region indicating higher detection sensitivity of new algal sp. Nevertheless, genus and species identification remains enigmatic due to conflicts between classical and molecular approaches. Despite some challenges, DNA barcoding may play a significant role in survey of marine biodiversity and prioritizing conservation strategies.

### 2.4. Marine Reptiles

When compared to fishes, there seems to be less data on DNA barcoding of reptiles. The first large-scale DNA barcoding of reptiles (including Squamata and Testudines) consisted of 468 samples from the biodiversity hotspot of Madagascar, which had identified 41-48 new (undescribed) species thereby showing the utility of DNA barcoding in biodiversity appraisal [47]. Likewise, it was uncovered that the average interspecific hereditary distance within families was 13.4% in Boidae and 29.8% in Gekkonidae [47]. In a study on Brazilian sea turtles, it was disclosed that species-specific COI barcode tags could be recognized in each of the marine turtle species that were explored [48]. In another study, DNA barcoding was performed on marine turtles which were globally threatened. This study had demonstrated that DNA barcoding is not only an influential tool for species discrimination but also can play an important role in wildlife forensics and conservational genetics [49]. No precise species number has been described for DNA barcoding for most marine reptiles. According to the report of the herpetofauna of Germany in 2016, the success rate of the identification of mitochondrial lineages representing species via DNA barcode was almost 100% because no cases of Barcode Index Number (BIN) sharing were detected within German native reptiles and amphibians [50].

### 2.5. DNA Barcoding of Marine Zooplanktons

Zooplanktons have incredible biological significance and represent 15 animal groups (phyla). In this manner, DNA barcoding of zooplanktons is a critical part of modern biological reviews. Census for Marine Zooplanktons (CMarZ) is dedicated to the investigation of worldwide zooplankton assemblages. The five DNA barcoding centres of CMarZ located at different parts of the world are Marine Science and Technology Center, University of Connecticut (USA), Alfred Wegener Institute for Polar and Marine Science, Bremerhaven (Germany), Ocean Research Institute, University of Tokyo (Japan), Institute of Oceanography, Chinese Academy of Sciences, Qingdao (China), and National Institute of Oceanography, Goa (India). Barcode tag analysis by using the COI gene as applied to 52 samples of 14 species of chaetognaths could effectively segregate distinctive types of chaetognaths over the phylum. The average Kimura 2-parameter distance (K2P distance) within the species was 0.0145. Among the marine zooplanktons, the copepods are one of the most deliberately unpredictable and naturally critical groups with more than 2500 species. Several reviews have been directed on this assorted group. The existence of cryptic species is widespread among the copepods, which requires more DNA barcoding studies [51]. DNA metabarcoding has been proved as an efficient method for measuring the biodiversity; however, the process of initiating long-term DNA-based monitoring programs or integrating with conventional programs is only at its starting stages. Metabarcoding generally produces more detections than microscopy, and its sensitivity may make cross-contamination during sampling a problem [52]. A research was conducted with Neocalanus copepods using four specific marker genes, namely, COI, 12S, nuclear ITS, and 28S [53]. Results had demonstrated that all four markers could recognize all species, but the distinction of the shape variation was just confirmed by COI sequences.

## 3. DNA Barcoding in Mammals

Mammals represent a distinct group of species for whom DNA barcoding was first proven to be successful, and this had primarily involved human hair as the test mammal primers [2, 4]. The primary usage of DNA barcoding ranges from the evaluation of the central web for morphologically mysterious species including carnivores, to capture misidentified individuals using hair, skin, and feathers and to reconsider critical descriptors with regard to food web framework [54–57]. Mitochondrial COI is mainly acknowledged as a standardized species level barcoding region in animals [58]. Mitochondrial genes are favoured over nuclear genes in case of mammals because mitochondrial genes do not have introns, and they are generally haploid and have less rate of recombination [2, 4, 59]. At first, 650 base fragments of the COI gene have been used, but later, a 100-base fragment of the original barcode is depicted as a useful fragment for all eukaryotes since it can be utilized in combination with next-generation sequencing to obtain a barcode of thousands of species immediately. COI as DNA barcoding region in animals much helps to recognize the species morphology without the presence of tangible attributes [60, 61]. Faeces from different species such as bats are used for successful identification by DNA barcoding [62]. By now, 2850 mammal species have been recorded by barcoding. If there are 7000 known species of mammals, then just 45% of those have been discovered reliably through succeeded barcoding [63, 64], and an estimated figure is depicted in Figure 2. For mammals as in humans, gene-level identification is a complex task due to the phylogenetic reconstruction delay [64, 65] and somehow due to sexuality which suppresses the genes of identification. Furthermore, the error rate can be increased due to the lack of voucher specimens and somehow caused by inaccurate taxonomy [66]. All techniques utilized for recognition have few disadvantages, yet COI has fewer disadvantages because of low indels; this is a helpful and reliable tool for identification in animals. DNA barcoding gives an operational system to mammalian taxonomic identification and cryptic species disclosure. Focused efforts to manufacture a reference library of genetic information has brought about the assembly of more than 35000 mammalian COI sequences and laid out the extent of mammal-related barcoding projects [67]. Besides, 5500 more extant mammalian species are currently recognized, and it will be impossible to achieve an ambitious goal without the active involvement of many institutions and experts worldwide. Therefore, a campaign has been launched that seeks to assemble a broad global coalition of leading researchers, museums, and other institutions with interest in mammal taxonomy and biodiversity (http://www.mammaliabol.org/).

## 4. DNA Barcoding in Insects and Birds

DNA barcoding utilizes a standardized region of the cytochrome c oxidase I (COI) gene to identify specimens at the species level. It has been proven to be a useful tool for the identification of avian samples [68]. It is also suggested to be a promising tool in conservation research due to its advantages over traditional species identification [69]. Because of expanding universal business and financial advancement, the annihilation rates and the introduction of invasive and pests species are growing [2, 4]; therefore, national and international client population is demanding faster species distinguishing proof and more data about their biodiversity. Along these lines, DNA barcoding is the best and quicker standard for insects, birds as well as for reptile species identification [70]. DNA barcoding offers favourable circumstances inside the cultivating divisions with the forecast of noteworthy bugs and intrusive assortments related to the checking of contaminated vectors which incorporate weird flying bugs [71]. For the most part, the COI gene is considered to be a barcoding region in most species of creepy crawlies and winged animals for their recognizable proof. By incorporating DNA barcoding with field observations in ecology, one can perceive new species of insects and birds effortlessly [72, 73]. For instance, skipper butterfly and cryptic species of hispine beetles can be recognized by linking the adult with larvae utilizing barcoding [20, 74]. It does not require any traditional morphological characters as for roots or juvenile bugs [6]. Mitochondrial COI-5′ and Mitochondrial COI-3′ regions are utilized to probe various sorts of creepy crawlies. DNA barcoding also permits the species identification of immature specimens which cannot be related to morphological characters [63]. A few of earthworm varieties have been recognized by utilizing the barcoding region at the larval stage [75]. For the identification of mosquito species, DNA barcoding has been proven to be a very robust tool to complement taxonomy [76–78]. Likewise, the COI-based DNA barcoding nearly attained a 100% success rate in recognizing the mosquito species [79, 80]. *COI* indicates that the quick rates of nucleotide substitution give an extraordinary scope of phylogenetic signals and help in revealing phylogeographic structures within species. Primers that are used for *COI* are universal as they are incredibly robust. Other mitochondrial protein-coding regions sometimes show extended indels (insertions/deletions) and are not recommended [2, 4]. Because sequence data on some of the insects' species is not accessible in databases, those insects require rapid identification with additional advanced tools [81]. It is estimated that about 643 types/species of North American birds were investigated fundamentally by utilizing the BirdF1 and BirdR1 primers. If the amplification was not fruitful, then different primers (FalcoFa, BirdR2, or vertebrate R1) were used [82]. Other regions of the mitochondrial CO1 gene have been reported to be a promising universal identification marker for barcoding of a bird's life. Almost 94% of these species have been analyzed while the remaining 6% are in the discussion thus far [83]. Over 2,597 waspsspecies belonging to six genera of Microgestin were barcoded from the tropical forest, rain forest, and cloud forest in northwestern Costa-Rica systematically via the use of DNA barcoding [84]. Considering the evidence across the experimental works discussed above, we can infer that DNA barcoding is reliable for most (94%) species including insects, birds, and others (see Figure 2). In the lingering 6%, barcode clusters related to small sets of firmly related species are known to hybridize in regular interval.

## 5. DNA Barcoding in Amphibians and Reptiles

In the past few years, environmental DNA (eDNA) and metabarcoding have inaugurated new avenues towards biodiversity studies. Amphibians and reptiles are animals for which these new avenues have opened up great leaps forward. There is growing evidence that eDNA can be proved to be potentially useful for studying terrestrial organisms for the evaluation of the relative abundance of species and the detection of reptiles [85]. Amphibian and reptile species are regularly changing (morphogenesis) and firmly different and contain profound nonspecific lineages which may prompt issues in species task with inadequate reference databases. Rather than various other taxa, mainly fishes and birds among vertebrates, DNA barcoding of amphibians and reptiles is at its early stages. The term amphibians are used to include all Lissamphibia, i.e., frogs, lizard, and caecilians (as of February 2012, totalling 6,922 species: 6,115 frogs, 618 lizards, and 189 caecilians) [86]. Reptiles are a paraphyletic gathering, where we utilize the term here to incorporate all nonavian surviving taxa of the Testudines, Crocodylia, Sphenodontia, and Squamata (as of February 2008, 8,734 species: 313 turtles, 23 crocodiles, 2 tuataras, and 8,396 squamates) (Uetz2010). DNA barcoding of reptiles, as a rule, is exceptionally restricted. Exceptional cases are the reasonable few types of marine turtles with high conservational suggestions, where a good advance of DNA barcoding was recently accomplished [48, 87]. In recent time, DNA barcoding is applied to recognize species intended by bush meat execution and to distinguish among other alligators and crocodiles [88, 89]. In amphibians, few test instances of COI DNA barcoding have been published [90–92] and a broad DNA barcoding project is currently being conveyed out on Central and South American taxa and has already led to extraordinary outcomes [93]. Until 2010, the broad majority of amphibian and reptile COI sequences were not originated in the frame of the global DNA barcoding strategy. Still, they are primarily the result of phylogenetic or phylogeographic work where COI was worn as one of the genetic markers [94]. Beyond considerations on DNA barcoding and phylogeny, there is an emerging number of mitogenomic appraises that have contributed COI sequences, among the ones with intense impact or including diverse species for reptiles [95–101] and amphibians [102–105]. These investigations have contributed to the number of available COI sequences but are otherwise not associated with the DNA barcoding effort as such. The general description of higher taxa, for example, orders and families in mitogenomic studies, is of vital significance because it permits the outline of primers for an assortment of the locale of the mitochondrial genome [106].

## 6. DNA Barcoding in Plants

**Figure 3:**
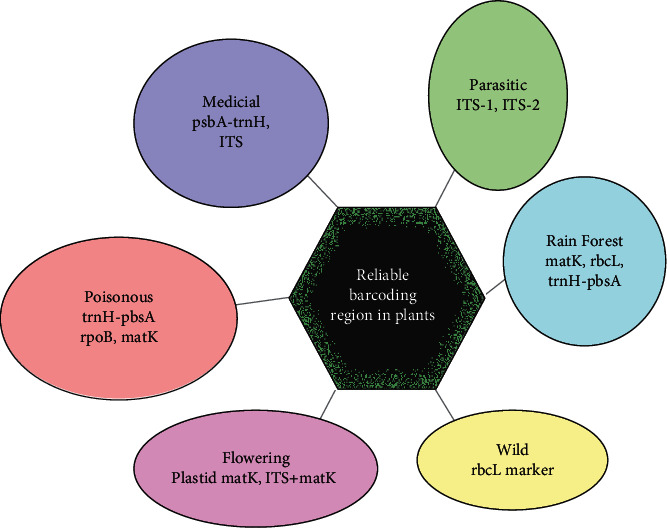
DNA barcoding in plants using different barcoding regions as a genetic marker.

Plants are excessively complex than animals; thus, barcoding in plants at the species level is at a debating level that had started ever since the last decade up until now [107–110]. However, barcoding in plants is relevant for distinguishing plants for whom there is no need of species-level identification (i.e., grasses and pine tree) [13, 111–113]. Mitochondrial genome in plants is predominantly replaced by the chloroplast genome due to the low rate of nucleotide substitution in plant mitochondrial genomes [114, 115]. However, in plants, several DNA barcodes have been proposed such as psbA-trnH intergenic spacer region [116], ITS2 region [80], matK gene, rbcL gene [117], trnL intron [118], ITS, and *trnL-F* intergenic spacer. Several noncoding plastid regions, for example, *psbA-trnH* intergenic spacer coupled with the trnL intron along with the internal transcribed spacers of nuclear ribosomal DNA, can also be employed. ITS has offered ancillary loci in individual tasks [118]. Moreover, these proposed barcodes involved various combinations of seven plastid markers. These included *rpoC1+rpoB+matK or rpoC1+matK+trnH-psbA* [119], *rbcL+trnH-psbA* [83], and *atpF-H+psbK-I+matK* as shown in Figure 3.

Using a two-locus blend of rbcL+matK as the standard DNA barcode has demonstrated a conviction strength of nearly 70%, as recently proposed by the Consortium for the Barcode of Life (CBOL) Plant Working Group (PWG) for the identification of plants [120, 121] whereby the reliability has been presented in Figure 2. Different barcode regions for plants are presented in Table 2.

Finding a plant equivalent has proven to be complicated. All barcodes presented in Table 1 are not in existence now. Many researchers have accepted that multiple markers will be required to obtain adequate species discrimination. A historical overview of the search for a reliable plant barcode is summarized and discussed briefly as follows.

### 6.1. Medicinal Plants

According to surveys in China, medicinal plants belong to 11,146 species from 2,309 genera of 383 families, thereby representing rich biodiversity [122]. For the identification of medicinal plants, the barcoding regions are specific. According to different researches, the barcoding region for medicinal plants is “psbA-trnH intergenic spacer” [125]. The Smithsonian group has developed a barcode library for 750 medicinal plants. However, Pennisi has been using yet another barcode combination to catalogue Chinese medicinal plants. With a probability of 72%, they discriminated over 907 samples from 550 species at the species level. Furthermore, [122] suggested that the second internal transcribed spacer (ITS2) of the nuclear ribosomal DNA represents the most suitable region for DNA barcoding applications. Furthermore, they tested the discrimination ability of ITS2 in more than 6600 plant samples belonging to 4800 species from 753 distinct genera and found that the rate of successful identification with the ITS2 was 92.7% at the species level [122].

### 6.2. Wild Plants

The barcoding region for wild plants has been identified by different research works. The barcode that is specific for wild plants is the rbcL marker, which is used for the identification of wild plants [123]. The use of rbcL gene sequences for diverse families of wild plants belonging to arid regions enabled the identification of a majority of samples (92%) to genus level and only 17% to species level. Research showed that this region has a high variation and identification ability.

### 6.3. Flowering Plants

Different research works had explained barcodes for the flowering plants. Lahaye and colleagues analyzed 1084 plant species (nearly 96% orchid species), and they had identified the portion of the plastid *matK* gene as a universal DNA barcode for flowering plants [126]. Another barcode region is also used for flowering plant genera, and that is the combination of ITS+matK [127]. By using this method, Kim and colleagues had barcoded 500 Korean flowering plant species, including dandelions, lilacs, and Cardamine.

### 6.4. Poisonous Plants

Five DNA barcode regions were evaluated for their identification of poisonous plants, and this contained three cpDNA sequences (trnH-psbA, rpoB, and matK) and two nuclear regions (at 103 and sqd1). In different works, the combination of matK with a nuclear marker such as at 103 is used to distinguish toxic plants [128]. This barcode also has the importance in the identification of cryptic orchid species.

### 6.5. Parasitic Plants

For the identification of parasitic plants, three barcode DNA regions were proposed, and this consists of rbcL, matK, and ITS. Different studies had concluded that the barcode for identification of parasitic plants as Pterygiella species is the ITS region, especially ITS-1 and ITS-2 [129]. Almost all species were delimited according to a phylogenetic analysis of ITS-2 sequences, ITS region, specifically ITS-1 and ITS-2 effectively discriminated all species in the genus when circumscribed Pterygiella according to phylogenetic and morphological analysis. By contrast, matK recognized only one clade (33.3%), whereas rbcL and matK+rbcL still failed to identify any clades.

### 6.6. Rain Forest Plants

The Atlantic Forest is the second-largest tropical forest in South America, with an original coverage of ~1.5 million km^2^ [130]. The Atlantic Forest is considered to be a hotspot of biodiversity [131], and it is comprised of highly diverse plants with an estimated 16,146 species, of which 7,524 are endemic. Among the taxa occurring in the Atlantic Forest that encounter difficulties for species identification is the Sapotaceae. This family consists of 53 genera and approximately 1,250 species with a pantropical distribution whereby most of which are found in tropical rainforests [124]. For the identification of forest plants, four plant barcode markers are evaluated as matK, rbcL, trnH-psbA, and the nuclear ribosomal internal transcribed spacer region—ITS. Different research studies suggested that the best barcode for forest plants is the ITS [124]. Notably, from an evaluation of over 80 samples from 26 species of Sapotaceae occurring in the Atlantic Forest, ITS yielded the highest average interspecific distance (0.122), followed by trnH-psbA (0.019), matK (0.008), and rbcL (0.002). These results indicate that the ITS region is the best option for the molecular identification of Sapotaceae species from the Atlantic Forest [132–134].

### 6.7. Limitation and Future Horizons of Barcoding in Plants

Pseudogenes and hybridization are the main problems of controversy in plant identification using barcodes [111, 135]. Researchers who did work in barcoding claim that taxonomy about phyla or specie is not universal which vary up to 50% that indicates the random rate of evolution [2, 4, 13]. This insists on being in touch with discoveries for future advancements to get command on this era. It had been surveyed that all primer sets have a range of functions; therefore, an appropriate solution may be there to use more than one primer combination [136]. Some biological phenomena that potentially interfere with barcoding are heteroplasmy, paternal leakage, introgression, polyploidization, recent speciation, incomplete lineage sorting, error in specimen identification, and incorrect taxonomy. These phenomena occur at different degrees depending on the dataset [2, 4, 25, 31]. So, it is essential to have a more significant number of data on individual species for the correct identification of species through traditional morphology as well as uploading correct online sequences to achieve robust barcoding for identifying different species. Kress and his collaborators are barcoding the 300 tree species in a long-term study site in Panama, and they planned to apply the same approach at 16 other study sites around the world. Moreover, Chase's group is developing a barcode database of endangered tropical trees for the United Kingdom to use in detecting illegal timber imports.

## 7. DNA Barcoding in Microorganisms

Barcoding is the main diversion in the field of microorganisms that cannot be segregated and evaluated in the research centre for phenotypic tests [14]. Barcoding in different types of microorganisms has diversity and versatility.

### 7.1. DNA Barcoding in Bacteria

The 16S ribosomal RNA and *Tuf* genes are utilized eminently as barcoding regions in bacteria [137]. The RIF marker framework incorporates a DNA-based replication initiation factor (RIF) that provides a significantly improved sequencing success. Also, a higher number of barcoding sequences rather than (internal transcribed spacer) ITS should extend to numerous bacterial genera, such as *Pseudomonas* [138]. Type II chaperonin (ortholog of cpn60) along with 16S rRNA was discovered as a choice for archeal detection in terms of Wolbachia [84, 139]. COI gene appeared to be a commonly used marker to provide the DNA barcode about 22 pathogenic species [140]. Similarly, chaperonin-60 (cpn60, also known as GroEL and Hsp60) is typically a successive bacterial barcode as a molecular chaperone preserved in bacteria [139, 141]. According to experts, the *rpoB* gene can also be used as a recognition marker gene for bacteria [142]. It is well researched that 16S rRNA, Tuf gene, and chaperonin along with COI could be the best bacterial barcoding regions, as shown in Table 3. In oceans, microbial growth causes the occurrence of nearly 98% of biochemical cycles, but DNA barcoding of microbial diversity has been poorly studied thus far. The rising community genomics and the metagenomics approaches may assure great bits of knowledge on prokaryote biodiversity and molecular evolution [39, 143–147]. Simultaneously, stepping up the enrollment of reference barcode sequences to apply high-throughput DNA barcoding to genus or species level identification in biodiversity research is also required [148].

### 7.2. DNA Barcoding in Fungi

For the *mitochondrial gene region, COI is a barcode for many organisms and was first discovered as a default marker by the Consortium for the Barcode of Life for all gathering of life forms, including fungi* [138]*. Research has shown that* ITS is the total and as often as a possible sequenced hereditary marker for fungi as in oomycete [1]. ITS is generally valuable for species identification in many fungal lineages and already functions as a *de facto* barcode [1, 3, 149]. *But, by default, COI marker is more dependable in a few clades of firmly related species such as in Penicillium, where it performs perfect, but in some other couple of fungal groups, it may often require cloning* [3, 65, 150]. Currently, there are ~172,000 reasonably full-length fungal ITS sequences in GenBank corresponding to ~15,500 species and 2,500 genera of fungi, generated from >150 countries on all continents and produced from ~11,500 scientific studies from ~500 different scientific journals. These data suggest 56% reliable identification with a Latin binomial, as mentioned in Figure 2. Thus, ITS has been developed as a predominant marker for DNA barcoding in fungi; however, it cannot recognize firmly related organisms or closely related other species. Sequence correlation of the ITS region is broadly used in taxonomy and organic molecular processes as a result of its ample ability to be amplified even from tiny quantities of DNA (due to the high copy variety of rRNA genes), thus a high level of variation even between closely related species. Therefore, it is considered to be slightly reliable.

### 7.3. DNA Barcoding in Viruses

Potentially, viruses are classified as the most primitive neurological organisms on the planet. It is frequently predicted that the fact that the count associated with viral allergens is more than 10 times that of the whole range of cells. The molecular assortment of viruses is challenging as the “molecular entity” along with virus type even now continue to be as an argument for many experts. The detection and also description concerning molecular agencies of the virus should be an essential purpose of DNA barcoding. Hardly few are induced to recognize pathogenically critical viruses. Consequently, experts are attempting to obtain barcodes for the discovery or recognition of viruses [151]. Wei and colleagues identified the particular *k*-mer-based barcode picture to find critical pathogenic human enteroviruses (HEVs) [152]. Through this practice, the fitness of 1 < *k* < 7 for any present *k* alongside a genome barcode appeared to be characterized concerning the k-mer consistency flow throughout the entire genome for many blends of *k*-mers. Bluetongue virus (BTV) is undoubtedly a pet virus that influences various animals such as domesticated animals, buffalo, sheep, deer, and goats [153]. To predict BTV occurrences in field settings, ultrasensitive approach, the Bio-Barcode Amplification Assay (BCA) strategy are being widely emanated. This approach was connected to the specific visualization of the outer-core protein VP7 with regard to BTV. Combining BCA with biosensor technique could provide ultrahigh detection sensitivity for analyzing protein and nucleic acids [154]. To distinguish avian influenza virus (AIV), a fluorescent DNA barcode-based immunoassay has been originated using the implementing sandwich immunoassay and fluorophore-tagged oligonucleotides as specialist barcodes [155]. To comprehend the viral biodiversity, continuous development of DNA or RNA-based methods can accelerate and facilitate viral research. On the other hand, it will be an ideal opportunity to elucidate the viral biodiversity with DNA barcoding, as there is no instance of reliability up until now for viruses.

## 8. DNA Barcoding and Food Products

DNA barcoding is attaining crucial importance in food authenticity studies due to its sensitivity, accuracy, and reliability in the identification of adulterants or adulterant species from pure food commodities [156]. Besides, barcoding is a productive technique for food supervision. Mainly, seafood is considered as a crucial part of the human diet. DNA barcoding can help extraordinarily to address the issue of mislabeling species. Yan et al.'s study has shown a practical use of DNA barcoding for preventing the extortion in international trade [157, 158]. It also has extraordinary significance to recognize cooked or processed fish. The prediction about the development of an individual diet from its waste items is now applicable by utilizing the DNA barcoding; a notable example is a fluid feeder [159]. The barcoding framework along with the assistance of parallel pyrosequencing had additionally been used to open up or analyze the diet of winged creatures, creepy crawlies, mollusks, and even mammals [160]. Barcoding is mostly used to distinguish formative changes in diet as per condition and investigate intraspecies interactions. DNA barcoding serves helpfully for beekeepers in obtaining honey bee items with particular nutritious or therapeutic characteristics as desired by the demands of the food market [161]. To identify diet and food items, mitochondrial COI in creatures has attained success even at the global level [162]. As DNA is available all around, thus, we can utilize DNA sequencing techniques by adding it to metabarcoding or NGS also called as “High Throughput DNA Sequencing.” NGS exhibits the capacity to distinguish organisms even from the complex mixture of faeces present openly in the field [56, 163–168]. With a specific end goal to recognize the living beings that are not present in either database, it is desired to look at firmly related organisms as to obtain a genus-level identification. At the point when reclamation identifies an organism not previously incorporated in the databases, it should then be submitted so that different scientists will have access to them. BOLD acts as the universal starting point for the identification of species, which would convey clients to refer to particular databases, for instance, pathogenic strains, endangered species, and disease vector species [169], which is used to create spate databases for various life forms yet at the same time most developed database framework is required independently for all organism which will be easy to handle by ordinary people especially databases related to insects and plants to achieve perfection in this field.

## 9. DNA Barcoding in Dentistry and Medical

So far, utilization of DNA barcoding in dentistry is making its way forward in future, with scarce studies yet. Recently, bacterial communities of oral cavity from children with dental caries have been identified using DNA barcodes. Ling et al. used high-throughput barcoded pyrosequencing along with PCR-denaturing gradient gel electrophoresis (DGGE) to investigate oral microbiota in supragingival plaques and saliva from 60 children (aged 3 to 6 years old) with and with no dental caries from Republic of China. The multiplex barcoded pyrosequencing was utilized in a single run using multiple of samples tagged solely via unique multiplex identifiers. As PCR-DGGE analysis is considered as a conventional molecular ecological approach, this analysis was also performed on the same samples, so the results of both approaches can be compared. A total of 186,787 high-quality sequences were investigated with the oral microbiota in children found to be far more diverse than previous studies reported, reporting 200 genera belonging to ten phyla in oral cavities. The results were more precise with diverse findings than conventional techniques used. Thus, it can be predicted that in future, DNA barcoding in dentistry can make its way with far excellence with more appropriate accuracy [170]. It can be anticipated that DNA barcoding in future can be utilized in pathological studies of samples from cells to diverse microbe identification towards DNA analyses, ultimately a way forward in medical forensic science with broader level of revolution.

## 10. DNA Barcoding and Taxonomy

There is significant contention concerning taxonomic point of view of molecular data, including DNA barcoding [171]. There are two foremost issues: (i) species identification and (ii) species disclosure. These are sometimes befuddled species identification by utilizing barcodes, which rely on the number of delegates of every species incorporated into the database. The most reliable approach is to acquire a DNA barcode that precisely shows a species at its base on the spectrum of that species. The principal depiction of new species utilizing a DNA barcode from the holotype was previously demonstrated [172] whereby the authors had used this technique to depict other types of xenothictis (Lepidoptera and Tortricidae). Since then, numerous new species have been identified with DNA barcodes from the holotype or paratypes, in arthropods as well as in other creatures [173–177]. On the contrary, species discovery is characterized as the taxonomic procedure of perceiving a large quantity of individual and additionally populaces as solitary animal categories. Thus, DNA barcode can accelerate species revelation. Firstly, DNA barcoding can be used to distinguish the cryptic and previously neglected species [108]. Secondly, DNA barcode data sorts all specimens of related taxa, notably when taxonomic investigations of these taxa are lacking [90, 178–180]. In museum collections, DNA barcodes can effectively flag errors [181]. However, it should be noticed that DNA barcoding cannot distinguish all the applicants of unidentified species, particularly for groups displaying genetic saturation.

## 11. Recent Evolutionary and Ecological Research with DNA Barcodes

A primary objective of evolutionary scientists and ecologists is to comprehend the origin of species and variables causing the imbalance in species abundance in various biomes across the globe. In many cases, the full spectrum of species in a given area is yet to be fully revealed, particularly in the most biologically diverse natural surroundings [182]. DNA barcodes have been incredibly valuable in the disclosure of cryptic and formerly unrecognized species of animals. For insects, it has been observed that new species can be uncovered through a combination of environmental field perceptions and DNA barcode markers [72, 73]. The use of DNA barcodes for the revelation of new species is developing to be a useful tool to clarify species boundaries and to measure the species assorted variety [183]. Similarly, the potential prevails for new plant species to be found and described as a result of hereditary inventories in light of both plastid and nuclear DNA barcode markers. For instance, in the involved tropical plant family Lauraceae, the group phylogeny created for the tree species on Barro Colorado Island with DNA barcode sequence information aided the identification of a formerly undescribed, however, suspected, new species of Nectandra [113]. Besides, a progressing DNA barcode study of trees in a forest dynamic plot situated in the heart of Amazon close to Manaus, Brazil, proposed that numerous “morphospecies” perceived by local taxonomists not currently to have scientific names may have compatible support from the DNA barcode sequence information. This hyperdiverse study plot in the Amazon of more than 1400 types of trees will possibly be an experiment for the utility of DNA barcodes in identifying species and discover novel ones.

## 12. DNA Barcoding Discourse and Future Challenges

A short mitochondrial gene encoding CO1 has been established as a standardized DNA barcode for many groups of animals. Similarly, DNA barcodes for plants (rbcL, matK, trnH-psbA, and ITS), fungi (ITS) and bacterial species (16S rRNA, Tuf gene, and chaperonin) have been standardized. Even though CO1 has been popularized to be of great utility for species identification, caution has been advocated with its application to groups of animals including reptiles and amphibians. In some taxa, DNA barcodes were not found efficiently reliable as they were first proposed. Plants were especially problematic during the early stages of utilizing DNA barcodes due to lower rates of nucleotide substitution in mitochondrial DNA as compared with that of animals. Furthermore, the concern that DNA barcodes will give erroneous identifications or poor results because of the complications of ancestral hybridization, polymorphisms, and introgression certainly applies to both plants and animals. These complications can be particularly acute in some groups of plants in which hybridization is widespread, and pseudogenes in the nuclear genome are frequently shared. More synergetic work is required on taxa with extensive hybridization to verify whether DNA barcodes can successfully provide accurate identifications across all species, while it is also true that low species resolution with DNA markers is due in part to our imperfect and variable definition of species. The variation in the success of DNA barcodes across lineages suggests that the processes of speciation and rates of evolution are also not uniform. The prime application of DNA barcodes will continue to be the identification of unknown samples.

Recent advances in sequencing approaches have broadened the analytical potential of DNA barcoding for routine biomonitoring applications to an unprecedented scale [184]. The target of obtaining the DNA barcodes from all species on the planet had rapidly led to the development of a Consortium for the Barcode of Life (CBOL, http://barcoding.si.edu). Progress in sequencing innovation implies that sequences can now be obtained quickly and efficiently so that this barcoding endeavour appears both as conceivable and advantageous. By using the short DNA sequences to bring the more superior reliability to the identification of species, a move ought to be made to supplement the mitochondrial DNA-based barcode with nuclear barcodes. This would decrease the issue of reliance on a solitary character and help to distinguish the situations where mitochondrial DNA carries differently to the nuclear genome. The usage of NGS technologies in many DNA barcode investigations is being expanded to answer both basic and applied biological questions. In general, NGS are not only used for constructing and collecting DNA barcode reference libraries for a specific set of taxa but will facilitate the ability to show all representative sequences that are present in a complex mixture of species thereby leading to the mapping of these sequences to reference DNA barcode databases. Most molecular phylogenetic reviews routinely utilize various nuclear genes. Efforts are ought to subsequently be made to develop nuclear barcodes to supplement the barcoding region that is in current use. As the points of interest and restrictions of barcoding become apparent, it is clear that the maximum efficiency at species identification will be achieved by taxonomic approaches that integrate DNA sequencing, ecological and morphology studies [185]. DNA barcodes are proving to be useful as evidence in investigations of natural and human-made disasters as well as in criminal cases. For instance, a library of CO1 markers for birds is now routinely being used to identify unknown avian species involved in aeroplane strikes. Even if DNA barcodes are not uniformly successful for unambiguous identifications across the entire Tree of Life, but scientists are already adopting DNA barcodes as a tool in their respective fields. The applications of barcodes are only in their infancy but will eventually become a significant source of data for the growing DNA barcode library.

## 13. Conclusion

DNA barcoding is one of the unique ideas of genetics with numerous inventive traits that have undertaken continuous improvements in its wide array of applications in life science. Individual reliabilities of barcoding in different organism range from unicellular to multicellular is not yet 100% reliable. DNA barcoding with specific merits and demerits is still a project of science that requires further efforts to fine-tune it. Furthermore, to meet future challenges and consummate reliability, DNA barcoding combined with NGS will lead to tremendous growth (i.e., in light of the available sequence data) to improve taxonomy and classification. To complement the barcoding regions, efforts should be made to develop the nuclear barcodes for augmenting that of DNA barcodes. The DNA barcodes are at the beginning of its application in species discovery, and inferring ecological and evolutionary relationships between species is anticipated to turn into a standard identification protocol for different living organisms.

## Figures and Tables

**Table 1:** List of different software/databases being used for DNA barcoding.

Organism	Software/databases	Description
Marine life	Genbank (http://www.ncbi.nlm.nih.gov)	Genbank data enable molecular diagnostic application on thousands of fish DNA sequences.
	FISH-Bol (http://www.fishbol.org)	Stores DNA barcodes of all fishes and provides a powerful tool to enhance the interaction of fish species. FISH-Bol is a robust tool that contains short regions of DNA as barcodes, images, and geospatial coordinates of voucher specimens.
Mammals	MammaliaBOL (http://www.mammaliabol.org/)	A comprehensive reference library of DNA barcodes for the global mammal fauna
Insects	Korea Barcode of life (KBOL) (http://www.upr-info.org/ database)	Stores barcode data of vertebrates, invertebrates, land tracheophytes, and lower plants
Amphibians and reptiles	G-BOL, German Consortium for the Barcode of Life: (http://www.g-bol.de/)	Stores COI barcoding fragment for 2000 specimens of Germany. This covers about 60% of the spider fauna and more than 70% of the harvestmen fauna recorded for Germany.
	BFB, Barcoding Fauna Bavarica: (http://www.faunabavarica.de/)	Barcodes the entire fauna (and also other life) of the German state Bavaria and stores barcoding data over 11,000 of animal species
Animals	Consortium for the Barcode of life (CBOL) (http://www.barcodeoflife.org)	CBOL is a barcoding database around the world and manages reference sequences for almost all animal life.
	IBOL (http://www.ibol.org)	Recently, the barcode of life project introduced a new phase with a launch of the international barcode of life (IBOL) for identification of animals.
Plants	Quarantine Barcode of Life (QBOL)(http://www.barcodeoflife.org/psa/barcoding/QBOL.pdf)	DNA barcode database was developed to identify quarantine organism in support of plant health.
	Tree-Bol	Compilation of barcoding data of tree species led by New York Botanical Garden
	Grass-Bol	Compilation of barcoding data of grasses under the supervision of Adelaide University and University of British Columbia
Fungi	CBOL (http://www.barcodeoflife.org)	Develops standard protocol and constructs a comprehensive DNA barcode library
	Assembling the fungal Tree of life (AFTOL) (http://www.uprinfo.org/database)	Provides insight on fungi evolution
	Canadian Centre for DNA barcoding (CCDB) (http://www.dnabarcoding.ca/)	Offers access to species identification
	ITS database	Identifies sequences of Ectomycorrhizal and Basidiomycetes
	Mycobank (http://www.mycobank.org)	Online database that documents new mycological names and combinations
	European Consortium for the Barcode of Life (ECBOL) (http://www.ecbol.org)	ECBOL functions as an information and coordination hub for taxonomists in Europe.
Bacteria	BOLD (http://www.barcodinglife.org)	Registers reference barcode sequences to apply high-throughput DNA barcoding to genus or species level identification in biodiversity research
	Bio Barcode (https://en.wikipedia.org/wiki/Barcode_system)	Allows biological specimen DNA sequence data to meet international standards by providing specialized services
	QBOL (http://www.qbol.org)	Acquires DNA barcode data of important species of bacteria and other organisms to build an analytical tool for quarantine

**Table 2:** Different barcode regions in the studied plants.

Plant barcode	References
nrITS	[117, 119]
nrITS2	[122]
matK	[115]
rpoB	[119]
rpoC1	[119]
psbA	[117]
rbcL	[123]
trnH-psbA	[119, 124]
Trns-L	[118]
UPA	[43]

**Table 3:** Barcode regions reported in different life forms.

Barcoding regions	Examples
Animals (COI)	*Holothuria edulis*, *Leptorhynchoides thecatus*, *Pomphorhynchus tereticollis*, *Acanthocephalus lucii*, *Allolobophora chlorotica*, *Polymorphus brevis*, *Axiothella constricta*, *Deosergestes corniculum*, *Caprella andreae*, *Microcosmus squamiger*, *Microcosmus squamiger*, *Nucula sulcata*, *Leptoplana tremellaris*, *Synecdoche constellate*, *Bruchomorpha beameri*, *Cixius nervosus*, *Diopatra neapolitana*, *Andrena humilis*, *Andrena fulvida*
Plants (matK, rbcL, psbA-trnH, ITS)	Rhubarb, *Pueraria candollei*, *Butea superb*, *Mucuna collettii*, *Galpemia* spp., *Dendrobium* spp., *Angelica* spp., members of *Rhododendron* genus, *Lonerica* spp., *Solanum* spp., and various adulterants, e.g., Astragalus spp.
	*Scutellaria* spp. *Astragalus* spp., and adulterants matK, psbA-trnH, *Lamia ceaemat* K, psbA-trnH, various medicinal plants matK, psbA-trnH, *Sabia* spp., Pteridophytes
	Paris spp. and adulterants, *Senna* spp., *Smilax* spp., *Phyllanthus* spp., *Cistance* spp., *Vitex* spp., matK, *Sideritis* spp., matK
	*Boerhavia* spp., *Astragalus* spp., and *adulterants*, *Sedum* spp., *Astragalus* spp., and adulterants, *Rubus* spp., *Hypericum* spp., *Ochradenus* spp., *Rehmannia* spp., *Dipsacus* spp., *Dendrobium* spp., *Paris* spp., *Citrus* spp., *Ruta* spp., *Astragalus* spp., *Meconopsis* spp., *Orobanche* spp.
Bacteria (COI, rpoB, 16S rRNA, Cpn60, Tuf)	Wolbachia, *Streptococcus* spp., *Buchnera* spp., *Rickettsia* spp., *Ehrlichia* spp., *Xanthomonas*, *Lactobacillus johnsonii*, *Streptococcus* spp., *Candidatus phytoplasma* spp.
Fungi (ITS, RPB1, RPB2, 18S (SSU))	*Fusarium virguliforme*, *Colletotrichum* spp., *Aureobasidium* spp., *Pseudocercospora* spp., *Cantharellus cibarius*, *Tricholomaviridi olivaceum*, *Laccariavinaceo-avellanea*, *Ramariama culatipes* (LSU), *Ramaria rubella*, *Ramaria stuntzii*, *Gomphus floccosus*, *Gautieria otthii*
Birds (Mitochondrial COI-5′ and COI-3′)	Herring Gull Larusargentatus, 14 Lesser Black-backed Gull Larusfuscus Caspian, Iceland Gull Larusglaucoides, Glaucous Gull Larushyperboreus, Great Skua Stercorarius skua
Marine life (COI)	Carangids, Clupeids, Scombrids, Groupers, Sciaenids, Silverbellies, Mullids, Polynemids, and Silurids. malacostracan species, *Ampelisca eschrichti*, *Ischyrocerus anguipes*, *Neomysis americana*, *Spirontocaris spinus*

## Data Availability

All data are available within the manuscript.
